# The Vall d’Hebron Initiative for Parkinson (VHIP) cohort: a prospective, longitudinal, and observational study enrolling *de novo* Parkinson’s disease patients for biomarker identification and validation

**DOI:** 10.3389/fnagi.2026.1858685

**Published:** 2026-07-23

**Authors:** Daniela Samaniego, Silvia Enríquez-Calzada, Laura Domingo, Mar Armengol, Maria Camprodón, Laura Melgarejo, Mercedes Arrúe-Gonzalo, Maria Miret-Milian, Victoria González, Sara Belmonte, Sara Lucas-Del-Pozo, Helena Xicoy, Anna López Carrasco, Jordi Riera, Eddie Pradas, Pablo Castillo-Sánchez, Marta Montpeyó, David Montpeyó, Miguel A. Zapata, Miquel Vila, Marta Martínez-Vicente, Ariadna Laguna, Jorge Hernández-Vara

**Affiliations:** 1 Neurodegenerative Diseases Research Group, Vall d’Hebron Research Institute (VHIR), Instituto de Investigación Sanitaria Acreditado Hospital Universitario Vall d’Hebron (IIS IR-HUVH), Network Center for Biomedical Research in Neurodegenerative Diseases (CIBERNED), Barcelona, Spain; 2 Department of Medicine, Autonomous University of Barcelona, Barcelona, Spain; 3 Rare and Metabolic Diseases Unit, University Hospital Vall d’Hebron, Barcelona, Spain; 4 Neurology Unit, University Hospital Vall d’Hebron, Barcelona, Spain; 5 Innovative Therapies in Pediatric Neurology Research Group, Vall d’Hebron Research Institute (VHIR), Barcelona, Spain; 6 Ophthalmology Unit, University Hospital Vall d’Hebron, Barcelona, Spain; 7 Department of Biochemistry and Molecular Biology, Autonomous University of Barcelona, Barcelona, Spain; 8 Catalan Institution for Research and Advanced Studies (ICREA), Barcelona, Spain

**Keywords:** autonomic function, biomarkers, cohort studies, neuroimaging, Parkinson’s disease, visual function

## Abstract

**Background:**

Parkinson’s disease (PD) is a progressive neurodegenerative disorder causing a variety of motor and non-motor symptoms. To date, no disease modifying treatment exists, and diagnosis is based on the manifestation of the clinical motor symptoms, which occurs when the neurodegenerative process is already advanced. Studies designed to find diagnostic/prognostic biomarkers and pathophysiological pathways involved in disease onset/progression are needed.

**Objective:**

To describe the study protocol of the Vall d’Hebron Initiative for Parkinson (VHIP) Cohort, a prospective and longitudinal observational cohort study aimed at deeply phenotyping *de novo* PD patients, including carriers of PD-linked mutations. We anticipate this initiative will contribute to identifying biomarkers for PD risk, diagnosis, and prognosis, as well as to stratify disease subtypes and increase our understanding of pathophysiology at early disease stages.

**Methods/Design:**

Subjects are prospectively recruited by movement disorder specialists at the Vall d’Hebron University Hospital (VHUH). To date, the study cohort includes 174 subjects—consisting of 117 patients with *de novo* Parkinson’s disease, 17 individuals with REM sleep behavior disorder (RBD), and 33 healthy controls—and recruitment is still ongoing. After informed consent, subjects are evaluated in detail to primarily assess objectives within four major domains of PD: motor, cognitive-affective, autonomic function, and vision. This includes first an extensive clinical evaluation (i) recording demographic, personal history, lifestyle, and dietary habits and (ii) performing clinical scales covering motor and non-motor symptoms. Second, data from autonomic function and visual function tests, together with neuroimaging data, are acquired. And finally, a wide range of biospecimens are collected for biochemical and molecular assessments including the genotyping of all participants. Participants receive follow-up assessments at 2.5 and 5-year intervals.

**Results/Discussion:**

VHIP is the first study to establish a Spanish cohort combining clinical, imaging, biochemical and molecular data over time in *de novo* patients, including PD-linked mutation carriers. VHIP provides a unique opportunity to link pre-diagnosis disturbances to PD development, as well as to identify risk, diagnosis, and prognosis biomarkers.

**Clinical trial registration:**

https://vhir.vallhebron.com/es/investigacion/proyecto-vall-dhebron-iniciativa-para-el-parkinson-vhip.

## 1 Introduction

Parkinson’s disease (PD) is the second most prevalent neurodegenerative disorder after Alzheimer’s disease, affecting up to 1 in 100 adults over the age of 60 (Von Campenhausen et al., 2005) and expected to double in the next two decades because of our aging society (Dorsey and Bloem, 2018). PD causes motor and non-motor symptoms that affect the quality of life of both patients and caregivers (Jankovic, 2008). Despite its large social impact, our understanding of the basic etiology, pathophysiology, and phenotypic diversity is still limited, and only symptomatic treatment options exist. In addition, the actual diagnosis is based on the manifestation of clinical motor symptoms, which occurs when the neurodegenerative process is already advanced, and the misdiagnosis rate is high (Rizzo et al., 2016). Though PD is clinically defined by motor symptoms resulting from dopaminergic neurodegeneration in the substantia nigra, PD is currently considered a disease involving a variety of pathways and neurotransmitters that may explain, in part, the large range of motor and non-motor symptoms. The manifestation of these symptoms greatly differs between PD subjects, making PD a heterogeneous disorder in which different clinical subtypes might represent different etiologies with different prognosis (Deng et al., 2018; Ryan et al., 2019). In addition, some of the non-motor symptoms (i.e., gastrointestinal, sleep and olfactory disturbances) are frequent and disabling and often precede the onset of motor symptoms by years (Rizzo et al., 2016), therefore, early identification and proper management of these symptoms is of crucial importance to provide the best care patients deserve.

PD is a multifactorial disorder with an increasing number of genes and environmental risk factors identified, although these are still far from explaining the majority of idiopathic cases. Multiple monogenic forms for PD have been described over the last 2 decades (Deng et al., 2018). Variants in the *Leucine-rich repeat kinase 2* (*LRRK2)* and the *Glucosylceramidase beta 1* (*GBA1)* gene are recognized as being the most frequent cause and risk factor, respectively (Ryan et al., 2019; Tolosa et al., 2020). Despite the important role of genetic factors in PD, genetic testing of clinically diagnosed PD patients and their relatives is not part of the standard-of-care testing, hindering the identification of participants candidates for future gene-tailored clinical trials and the identification of participants as mutation carriers (MC) for risk/diagnosis biomarker discovery studies. Also, despite being key genetic factors involved in PD, their penetrance and expressivity are variable, and the genetic and environmental factors contributing to the manifestation and expressivity of pathogenic variants are not fully understood yet.

To advance our understanding of PD pathophysiology and accelerate the implementation of disease modifying treatments, it is essential to investigate PD considering its large clinical heterogeneity and the complexity of underlying mechanisms. Moreover, reliable and well-validated biomarkers to identify individuals at risk before motor symptoms manifest, to accurately diagnose individuals at the threshold of clinical PD, and to monitor PD progression throughout the course of the disease are essential for proper disease diagnosis, adequate classification of PD subtypes and disease management. To date, there are no reliable biomarkers for any of these purposes. In addition, biomarker studies should avoid the possible confounding effect of dopaminergic medication by including, as well, treatment-naïve PD subjects at baseline. Also, studies designed to identify risk and diagnosis biomarkers should include longitudinally followed non-manifesting MC in PD-linked genes and prodromal PD patients. However, such study cohorts are sparsely available DUPARC, LIPAD (Boertien et al., 2020; Usnich et al., 2021) and not existing in a large country as Spain. According to the most recent data, updated for 2025, from the Spanish Society of Neurology (SEN) and the Spanish Parkinson’s Federation (FEP), the disease’s impact in Spain remains significant, with an estimated prevalence over 200,000 affected individuals. Furthermore, the incidence of the disorder continues to rise, with approximately 10,000 new cases diagnosed annually across the country (Sociedad Española de Neurología, 2025).

We describe here the Vall d’Hebron Initiative for Parkinson (VHIP) study protocol, a single-center, prospective, longitudinal, observational, cohort study at the Vall d’Hebron University hospital in Barcelona (VHUH, Spain). Our cohort essentially includes newly diagnosed PD subjects, treatment-naïve at baseline, subjects at risk with isolated REM sleep behavior disorder (RBD), and healthy controls. After sequencing, subjects are then re-classified according to their genetic background. The VHIP study includes the evaluation of participants across a wide variety of domains, focusing most on four PD domains: (1) motor, (2) cognitive-affective (3) autonomic function and (4) vision. All participants are extensively characterized with the aim to identify biomarkers for PD risk, diagnosis, and prognosis, as well as to stratify disease subtypes and increase our understanding of pathophysiology at early disease stages, specially focusing in the preclinical research areas of interest and the expertise of our research group: (1) lysosomal function, (2) immune function, (2) gastrointestinal function, (3) vision (4) neuromelanin (NM) metabolism. Our effort to harmonize and standardize operational procedures with ongoing international cohort studies for comparability and cross validation reflects the willingness of the VHIP study to represent a national and international source for sharing and collaborative efforts in PD research.

## 2 Methods

### 2.1 Study design

**Figure 1:**
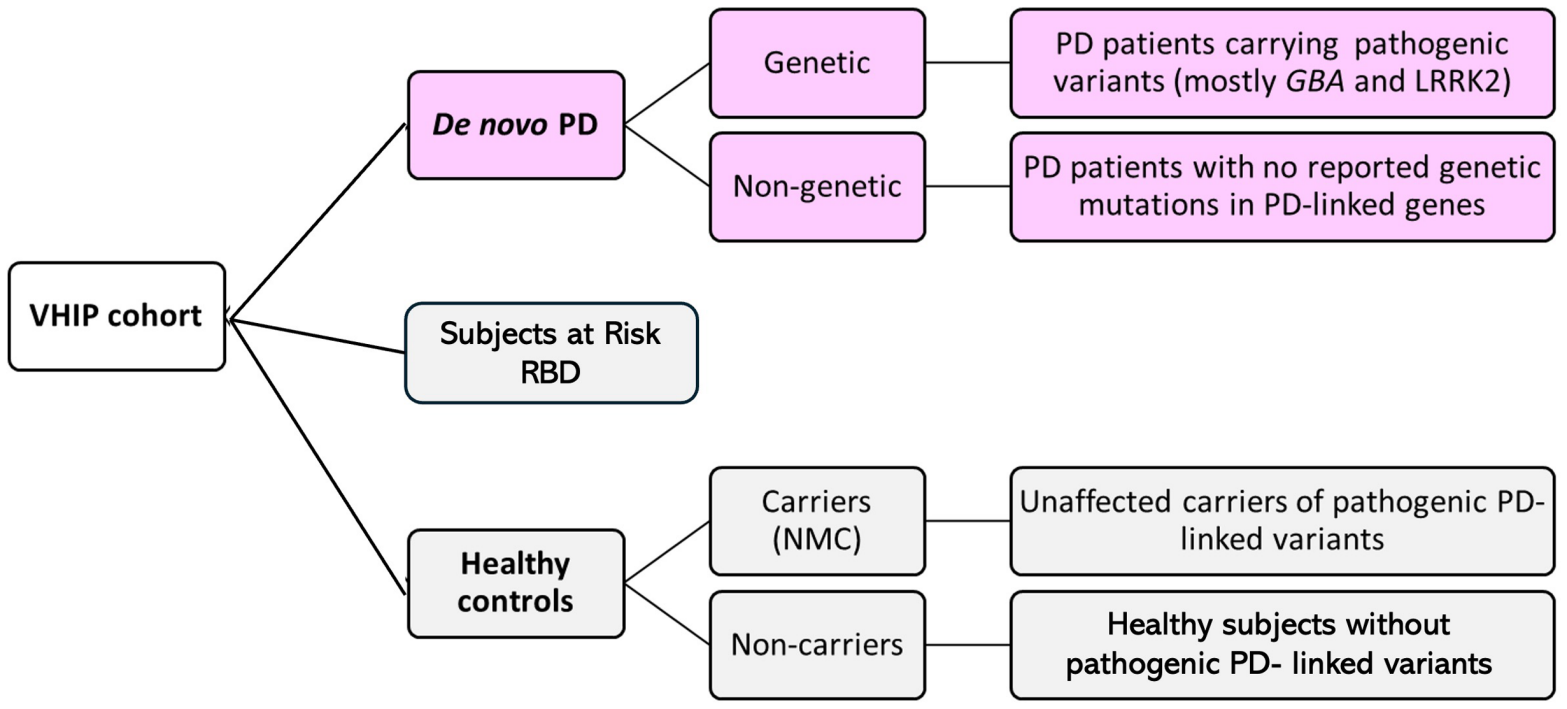
VHIP cohort participants. VHIP cohort study enrolls (i) de novo PD patients including genetic (PD patients carrying pathogenic mutations, mostly GBA1 and LRRK2) and non-genetic participants (PD patients with no reported pathogenic PD-linked mutations); (ii) individuals at increased risk of developing synucleinopathies, such as patients with isolated REM sleep behavior disorder (RBD); (iii) healthy controls, covering carriers (unaffected carriers of PD-linked variants) and non-carriers (healthy subjects without pathogenic variants). NMC: non-manifesting mutation carriers.

The VHIP study started in 2019 as a single-center, prospective, longitudinal, observational, cohort study that enrolls: (i) healthy controls; (ii) individuals at increased risk of developing synucleinopathies, such as patients with RBD confirmed by polysomnography; and (iii) *de novo* PD patients, treatment naïve at baseline. Subjects from each group undergo targeted NGS sequencing and are then re-classified according to their genetic background in genetic PD, idiopathic PD, non-manifesting carriers (NMC) and non-carriers healthy controls (Figure 1). Participants are recruited by movement disorders specialists at VHUH, which has the capacity to include 1-2 subjects/week to this study. All participants are age and sex-matched between groups. Although most of the participants recruited live in Catalonia, our center has been labeled CSUR (certification granted by the Spanish Ministry of Health to reference units with expertise in movement disorders), allowing the inclusion of patients living in other Spanish regions. After informed consent and the completion of the baseline assessments, PD patients continue care as usual, including the start of dopaminergic treatment when necessary. Healthy controls, mostly age- and sex-matched to NMC and manifesting PD groups, are recruited by the same movement disorders specialists. Healthy relatives from PD patients, who would like to participate voluntarily, and other healthy participants are being recruited and genotyped for PD-linked mutations to classify them as NMC or non-carrier controls. Detailed clinical examination, including motor and non-motor symptoms, cognitive-affective assessments, autonomic function, and visual measurements, combined with imaging data and biosample collection are performed at baseline visits with a follow-up after 2.5 year and with an extended follow-up visit at 5 years intervals.

### 2.2 Study goals and timetable

The VHIP cohort study is designed to assess different specific objectives with the ultimate goal of discovering and validating biomarkers for PD risk, diagnosis, and prognosis, as well as to stratify disease subtypes and increase our understanding of pathophysiology at early disease stages. The first primary objective is to perform an exhaustive clinical examination of participants recording demographic and lifestyle data, personal and medical history, complete physical and neurological examination accompanied by several standardized and validated clinical scales. The second is to obtain autonomic function, visual and imaging measurements from all subjects in the cohort. The third is to collect and store a wide-ranging repository of blood and other biological samples from each participant. Using these data and samples, some of the secondary objectives are: (i) to determine the frequency of motor and non-motor symptoms and correlate clinical variables with lifestyle factors, genetic and molecular biomarkers; (ii) to assess the potential of autonomic, visual and neuroimaging disturbances as clinical predictive biomarkers for PD; (iii) to study the relationship between PD manifestations and GBA1/lysosomal dysfunction; (iv) to study the relationship between the immune and gut dysfunction in the pathogenesis of PD; and (v) to develop an *in vitro* platform to facilitate personalized medicine and therapeutic screenings (Table 1). Future additional secondary objectives might derive from the obtained results or arise from other relevant research lines in the field.

**Table 1:** Objectives of the VHIP cohort study.

Overall objectives
To discover and validate biomarkers for PD risk, diagnosis, and prognosis.
To stratify disease subtypes based on clinical, genetic, and biochemical/molecular correlations.
To increase our understanding of disease pathophysiology at early disease stages.

Primary objectives
To perform an exhaustive clinical examination of participants recording demographic and lifestyle data, personal and medical history, complete physical and neurological examination accompanied with several standardized and validated clinical scales at baseline and at 2.5 and 5 years of follow up.
To obtain autonomic function, visual and imaging measurements from all participants in the cohort at baseline and at 5 years of follow up.
To collect and store a wide range of biospecimens including blood, cerebrospinal fluid, urine, feces, oral mucosa, olfactory mucosa and skin biopsies from each participant at baseline and at 5 years of follow up.

Secondary objectives
To study the frequency of motor and non-motor symptoms in the different subgroups of study and to compare it against a sex- and age-matched control group.
To study the association between lifestyle factors and clinical manifestations in prodromic and manifest PD patients compared to age- and sex- matched controls subjects.
To study the relationship between different clinical, genetic, molecular, and imaging variables in the different subgroups of study, and along the time of follow-up, to find correlations and identify/validate biomarkers.
To assess the potential of autonomic dysfunction as a clinical biomarker for PD, with a special focus on *GBA1* and *LRRK2* mutation carriers to identify autonomic symptoms assessments as potential risk biomarkers, as well as *de novo* and manifest PD patients (sporadic and genetic) with age- and sex- matched control subjects, to identify diagnosis and prognosis biomarkers.
To evaluate parameters related to visual function as a clinical biomarker for PD, with a special focus on *GBA1* and *LRRK2* mutation carriers to identify retinal alterations as potential risk biomarkers, as well as *de novo* and manifest PD patients (sporadic and genetic) with age- and sex- matched control subjects, to identify diagnosis and prognosis biomarkers.
To evaluate parameters related to neuroimaging as a clinical biomarker for PD, with a special focus on *GBA1* and *LRRK2* mutation carriers to identify brain alterations as potential risk biomarkers, as well as *de novo* and manifest PD patients (sporadic and genetic) with age- and sex- matched control subjects, to identify diagnosis and prognosis biomarkers.
To study the relationship between PD and GBA1/lysosomal dysfunction (i.e., genetic variants, enzymatic activities, and lipid metabolism) as potential biomarkers of PD risk, diagnosis, and prognosis.
To determine the contribution of immune and gut dysfunction to the pathogenesis of PD and their potential as biomarkers of PD risk, diagnosis, and prognosis.
To develop *in vitro* models based on human biosamples (i.e., PBMCs and fibroblasts from skin biopsies) as *in vitro* platforms to facilitate personalized medicine and therapeutic screenings.

Pre-start procedures started in 2018 and the first visit was performed in Q3-2019. To date (Q1-2026), the study cohort includes 174 subjects—consisting of 117 patients with *de novo* Parkinson’s disease, 17 individuals with RBD, and 33 healthy controls. Data review began Q1-2026, and the first analyses with the cohort data, detailed baseline characterization and cross-sectional clinical and biomarker data of the participants, are expected for Q3-2026. Recruitment is still ongoing to increase the number of participants.

### 2.3 Study population

Recruited participants have to meet the following inclusion criteria: (1) to be older than 18 years of age; (2) to provide written informed consent; (3) to be able to comply with study procedures; (4) for PD patients, clinical diagnosis of PD according to the Movement Disorder Society (MDS) Clinical Diagnostic Criteria for PD (Postuma et al., 2015); (5) for RBD patients, clinical and instrumental diagnosis of REM Sleep Behavior Disorder according to the International Classification of Sleep Disorders, 3rd Edition (ICSD-3) criteria, confirmed by video-polysomnography (v-PSG). Concurrently, healthy controls are recruited within the hospital setting, comprising patients’ relatives, caregivers, and individuals from the same hospital environment. The inclusion criteria for controls requires the absence of clinical signs or symptoms of Parkinson’s disease, absence of sleep disturbances suggestive of RBD, and an age-matched distribution relative to the study cohorts. On the other hand, participants will be excluded from the study if: (1) are not capable of completing clinical scales adequately; (2) have a diagnosis of a significant central or peripheral nervous system disease affecting the subject’s cognition or motor function at any time, such as another neurodegenerative disorder, multiple sclerosis or stroke; (3) have other severe and disabling concomitant non-neurological disease (oncological, autoimmune, etc.); (4) have known chronic anemia and/or hyperuricemia; (5) have dementia criteria according to DSM-V criteria (The Montreal Cognitive Assessment [MoCA] < 21 used as screening tool); (6) have a diagnosed gastrointestinal disorder affecting the composition of the gut microbiome; (7) if long-term follow-up is not expected to be possible or whose PD diagnosis is rectified during follow-up; (8) *de novo* PD subjects, prior use of dopaminergic therapy (e.g., levodopa, dopamine agonist, amantadine) for 30 or more days any time in the past. The same inclusion and exclusion criteria, except for PD diagnosis and therapy, are applied for the control groups.

### 2.4 Study procedures

**Figure 2:**
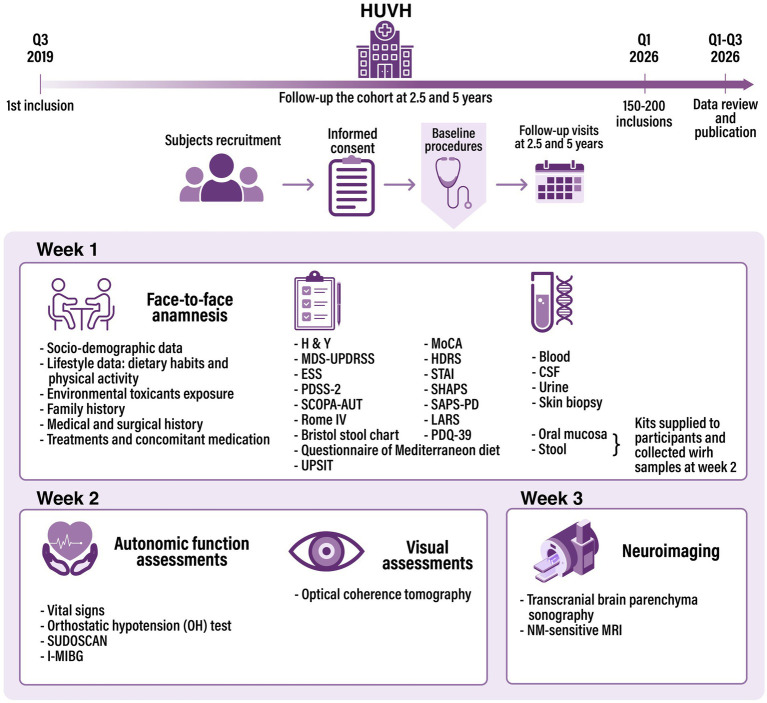
Flowchart of recruitment, baseline, and follow-up visits assessments in the Vall d’Hebron Initiative for Parkinson (VHIP) cohort.

After signature of the informed consent, the evaluations of the baseline visit begin with a total duration of 3 weeks (Figure 2). After 2.5 and 5 years, a follow-up visit is performed repeating some clinical scales and sample collection depending on the group and visit. Details of which assessment is performed in each visit are specified in Table 2.

**Table 2:** Assessments performed in the study, segregated by group and visit type.

Assessments	De novo PD and RBD groups						Control groups					
	Genetic PD/RBD			Non-genetic PD/RBD			Carriers (NMC)			Non-carriers		
	Baseline	2.5Y FUP	5Y FUP	Baseline	2.5Y FUP	5Y FUP	Baseline	2.5Y FUP	5Y FUP	Baseline	2.5Y FUP	5Y FUP
Face to face anamnesis	X	X	X	X	X	X	X	X	X	X	X	X
Clinical scales												
H&Y	X	X	X	X	X	X	X	X	X	X	X	X
MDS-UPDRS	X	X	X	X	X	X	X	X	X	X	X	X
ESS	X	X	X	X	X	X	X	X	X	X	X	X
PDSS-2	X	X	X	X	X	X	X	X	X	X	X	X
SCOPA-AUT	X	X	X	X	X	X	X	X	X	X	X	X
Rome IV	X	X	X	X	X	X	X	X	X	X	X	X
Bristol stool chart	X	X	X	X	X	X	X	X	X	X	X	X
Quest. of Mediterranean diet	X			X			X			X		
UPSIT	X		X	X		X	X		X	X		X
MoCA	X	X	X	X	X	X	X	X	X	X	X	X
HDRS	X		X	X		X	X		X	X		X
STAI	X		X	X		X	X		X	X		X
SHAPS	X		X	X		X	X		X	X		X
SAPS-PD	X		X	X		X	X		X	X		X
LARS	X		X	X		X	X		X	X		X
PDQ-39	X	X	X	X	X	X	X	X	X	X	X	X
Biosamples collection												
Blood	X		X	X		X	X		X	X		X
CSF	X		X	X		X	X		X	X		X
Urine	X		X	X		X	X		X	X		X
Skin biopsy	X		X	X		X	X		X	X		X
Olfactory mucosa	X		X	X		X	X		X	X		X
Oral mucosa	X		X	X		X	X		X	X		X
Stool	X		X	X		X	X		X	X		X
Autonomic function assessments												
Vital signs	X		X	X		X	X			X		X
Orthostatic Hypotension test	X		X	X		X	X			X		X
SUDOSCAN	X		X	X		X	X		X	X		X
I-MIBG*	X			X			X			X		
Visual assessments												
OCT*	X		X	X		X	X			X		X
Imaging												
Transcranial brain parenchyma sonography	X			X			X			X		
NM-sensitive MRI*	X		X	X		X	X		X	X		X

H&Y, Hoehn & Yahr Scale; MDS-UPDRS, Movement Disorders Society Unified Parkinson’s Disease Rating Scale; ESS, Epworth Somnolence Scale; PDSS-2, Parkinson’s Disease Sleep Scale; SCOPA-AUT, Scale for autonomic function; UPSIT, University of Pennsylvania Smell Identification Test; MoCA, Montreal Cognitive Assessment; HDRS, Hamilton Depression Rating Scale; STAI, State–Trait Anxiety Inventory; SHAPS, Snaith-Hamilton Pleasure Scale; SPAS-PD, Scale for Assessment of Positive Symptoms for Parkinson’s disease; LARS, Lille Apathy Rating Scale; PDQ-39, 39-item Parkinson’s Disease Questionnaire; *MIBG, Iodine-123 metaiodobenzylguanidine myocardial scintigraphy; OCT, Optical Coherence Tomography; NM-MRI, neuromelanin-sensitive magnetic resonance imaging are assessed in a selected subgroup of participants.

#### 2.4.1 Clinical examination (first week)

Collection through face-to-face anamnesis of extensive information on socio-demographic aspects, lifestyle data such as dietary habits and physical activity, exposure to environmental toxins, family history, factors related to PD, medical and surgical history, treatments, and concomitant medication during the last 6 months (1 h). Then, a physical and neurological examination is performed followed by a series of standardized and validated clinical scales (Duration: investigator-rated (1.5 h) and self-rated (1 h). We used validated versions of questionnaires and scales in Spanish as the local language (Table 3). The same evaluations done for PD subjects are performed in RBD and in control subjects unless those ones specific for PD in the latter case.

**Table 3:** Clinical scales assessed in the VHIP study.

Clinical scale	Measurement	Methodology	Evaluation	Bibliography
H&Y	Severity of PD symptoms	Descriptive staging scale to evaluate both disability and impairment related to clinical PD progression.	Neurologist	(Hoehn and Yahr, 1967)
MDS-UPDRS	Severity of PD symptoms	Evaluates motor and non-motor impacts of PD across 4 parts: part I, non-motor aspects of experiences of daily living; part II, motor aspects of experiences of daily living; part III, motor examination and part IV, motor complications.	Neurologist	(Goetz et al., 2008)
ESS	Daytime sleepiness	Assesses the likelihood of dozing off or falling asleep in daily common situations.	Neurologist	(Johns, 1991)
PDSS-2	Quality of night sleep	Addresses 15 commonly reported symptoms associated with sleep disturbance in PD	Neurologist	(Trenkwalder et al., 2011)
SCOPA-AUT	Autonomic symptoms	25-items scale assessing gastrointestinal, urinary, cardiovascular, thermoregulatory, pupillomotor, and sexual domains.	Self-completed	(Visser et al., 2004)
Rome IV	Gastrointestinal function	Categorizes disorders of chronic constipation into four subtypes: (a) functional constipation, (b) irritable bowel syndrome with constipation, (c) opioid-induced constipation, and (d)functional defecation	Self-completed	(Drossman and Hasler, 2016; Palsson et al., 2016)
Bristol stool chart	Gastrointestinal function	Classifies feces into seven groups to monitor bowel movements.	Self-completed	(Lewis and Heaton, 1997)
Quest. of Mediterranean diet	Adherence to Mediterranean diet	14-item tool measuring adherence to the Mediterranean diet	Self-completed	(Schröder et al., 2011)
UPSIT	Olfactory system	40 questions test evaluating the individual’s ability to detect odors at a suprathreshold level.	Self-completed	(Doty et al., 1984)
MoCA	Cognition	Measures language, visuospatial ability, memory, recall, and abstract thinking.	Neurologist	(Nasreddine et al., 2005)
HDRS	Neuropsychiatric symptoms: depression	Evaluates symptoms of depression experienced over the past week.	Neurologist	(Schrag et al., 2007)
STAI	Neuropsychiatric symptoms: anxiety	40-item self-report scale that assesses separate dimensions of “state” and “trait” anxiety.	Self-completed	(Hamilton, 1960)
SHAPS	Neuropsychiatric symptoms: anhedonia	14-item assessing hedonic capacity.	Self-completed	(Snaith et al., 1995)
SAPS-PD	Neuropsychiatric symptoms: existence of psychosis	Evaluates for hallucination-and delusion-type symptoms.	Neurologist	(Voss et al., 2013)
LARS	Neuropsychiatric symptoms: apathy	33 items scale detecting and quantifying apathy.	Neurologist	(Marin et al., 1991)
PDQ-39	Quality of life	Assesses health-related quality of life in people with PD.	Self-completed	(Jenkinson et al., 1997)

#### 2.4.2 Autonomic function assessments (second week)

The accumulation of misfolded α-synuclein (α-Syn) in synucleinopathies occurs in neurons or glia of both the central and peripheral nervous system, including the autonomic system (Kaufmann et al., 2001). To evaluate autonomic function, patients undergo a non-invasive test that measures how their nervous system works to control blood pressure, heart rate, and sweating.

##### 2.4.2.1 Sympathetic tests

The sympathetic branch is often affected early in PD due to postganglionic denervation.

###### 2.4.2.1.1 Orthostatic Hypotension (OH) test

The evaluation of Neurogenic Orthostatic Hypotension (nHO) in Parkinson’s Disease (PD) focuses on the failure of the sympathetic noradrenergic system to maintain blood pressure upon standing (Kaufmann et al., 2001; Palma and Kaufmann, 2020; Goldstein et al., 2005). According to the consensus nHO is diagnosed as a sustained reduction in blood pressure within 3 min of standing or head-up tilt, defined by a drop of at least 20 mmHg in systolic or 10 mmHg in diastolic pressure (Palma and Kaufmann, 2020; Goldstein et al., 2005). However, in patients with pre-existing supine hypertension—a common finding in PD—these authors recommend a more stringent threshold of 30/15 mmHg to avoid over-diagnosis. The symptoms in PD are not always “dizziness.” Patients may report “coat-hanger” pain (neck and shoulder ache), blurred vision, fatigue, or cognitive slowing, which can sometimes be mistaken for a levodopa “off” state. This assessment is performed on all subjects of the cohort after 10 min of rest in a supine position (Palma and Kaufmann, 2020).

###### 2.4.2.1.2 SUDOSCAN

A non-invasive method that uses reverse iontophoresis to evaluate the integrity of small sympathetic cholinergic fibers that innervate the sweat glands through Electrochemical Sweat Conductance (ESC) in the palms and soles (Freeman et al., 2011). The procedure is based on the principle of reverse iontophoresis and chronoamperometry to determine the integrity of the small sympathetic C-fibers that innervate the sweat glands. The patient places their bare palms and soles on large stainless-steel electrodes. A low-voltage direct current (less than 4 V) is applied to the electrodes. This current stimulates the sweat glands, causing chloride ions to be drawn from the sweat ducts toward the electrodes. The device measures the current generated by this movement, which is then converted into Electrochemical Sweat Conductance (ESC), expressed in microsiemens (μS). The entire test is completed in approximately 3 min. While specific thresholds can vary slightly by age and ethnicity, the general clinical standards are as follows: normal: ESC > 60 μS, moderate Dysfunction: ESC between 40 and 60 μS and severe dysfunction: ESC < 40 μS (Freeman et al., 2011; Ziemssen and Siepmann, 2019).

###### 2.4.2.1.3 Iodine-123 metaiodobenzylguanidine myocardial scintigraphy (MIBG)

A hallmark extracerebral feature of PD is postganglionic cardiac sympathetic denervation, resulting from the pathological deposition of alpha-synuclein within the paravertebral ganglia and myocardial nerve terminals. As a functional analog of norepinephrine, MIBG is internalized by presynaptic norepinephrine transporters (NET) in myocardial sympathetic nerve endings (Orimo et al., 2012). While cardiac innervation typically remains intact in healthy individuals and patients with atypical parkinsonian syndromes—such as Multiple System Atrophy (MSA) or Progressive Supranuclear Palsy (PSP)—PD patients exhibit a marked reduction in tracer uptake. Cardiac sympathetic denervation frequently precedes the onset of motor symptoms. Consequently, IMBG imaging can identify Lewy body pathology during the prodromal phase, such as in patients with REM sleep behavior disorder (RBD). The clinical value of this imaging modality is so robust that the International Parkinson and Movement Disorder Society (MDS) has formally included reduced MIBG uptake as a key supportive criterion for the clinical diagnosis of PD (Sociedad Española de Neurología, 2025).

After tracer administration, planar images are acquired 15 min (early) and 3-4 h (delayed) post-injection. The Heart-to-Mediastinum (H/M) ratio is calculated by comparing tracer uptake in the heart versus the mediastinum. Normal: H/M ratio >2.0 (standardized values may vary slightly by center). Abnormal (PD): Reduced H/M ratio (often <1.5). Low uptake is highly suggestive of PD and helps differentiate it from other parkinsonisms. Given availability and cost, this test is performed in our cohort in selected cases (Orimo et al., 2012).

##### 2.4.2.2 Parasynmpathetic tests

###### 2.4.2.2.1 Heart rate (HR) change

In neurogenic OH, the heart rate increase is usually blunted (less than 15 beats per minute) despite the large drop in BP. This is because the baroreflex is “broken” at both the sympathetic and parasympathetic levels (Kaufmann et al., 2001).

#### 2.4.3 Visual measurements (second week)

Optical Coherence Tomography (OCT) is a non-invasive imaging modality that utilizes low-coherence interferometry to provide high-resolution, cross-sectional visualizations of retinal morphology, serving as a reliable “*in vivo*” biomarker for neuroaxonal loss in neurodegenerative conditions such as PD (Navarro-Otano, 2014; Huang et al., 1991). In this study, ophthalmological examination of both eyes includes Best Corrected Visual Acuity (BCVA) via Snellen charts, color vision assessment with Ishihara plates, and pupillary reactivity analysis using AlgiScan (Idmed, Marseille, France). Clinical imaging consists of a 50 ° retinography and the measurement of Retinal Nerve Fiber Layer (RNFL) and Ganglion Cell Layer (GCL) thickness, which are frequently thinned in patients with PD (Zou et al., 2023; Campagnolo et al., 2025). Furthermore, OCT-Angiography (OCT-A) scans (4.5 × 4.5 mm) are acquired using the DRI OCT Triton (Topcon Corporation, Tokyo, Japan), where the Vessel Density (VD) of the superficial and deep layers is automatically segmented and the Foveal Avascular Zone (FAZ) area is manually delineated to assess microvascular integrity (Hajee et al., 2009).

#### 2.4.4 Imaging measurements (third week)

Neuroimaging techniques have transformed the clinical approach to Parkinson’s disease by providing objective biomarkers that allow for the visualization of neurodegenerative processes, facilitating both early diagnosis and the differentiation from other movement disorders.

##### 2.4.4.1 Transcranial brain parenchyma sonography (TCS)

TCS is performed at baseline in all participants to assess the presence or absence of qualitative substantia nigra hyperechogenicity. In addition, a bilateral semiquantitative assessment of hyperechogenicity is conducted. TCS is carried out using a color-coded B-mode ultrasound system equipped with a 2.5-MHz transducer (Toshiba Aplio 500®, Toshiba Medical Systems). Images are obtained through the preauricular acoustic window with a penetration depth of 16 cm, with signal brightness and time again compensation adjusted as needed to optimize image quality. Due to the stability of TCS findings over time, no repeat TCS is performed during follow-up visits (Berg et al., 2012).

##### 2.4.4.2 Neuromelanin-sensitive MRI (NM-MRI)

NM-MRI is a specialized neuroimaging technique that allows for the “*in vivo*” visualization of the substantia nigra pars compacta (SNc) and the locus ceruleus (Barrett et al., 2013). By utilizing the paramagnetic properties of neuromelanin-iron complexes, this modality provides a direct assessment of the loss of dopaminergic and noradrenergic neurons, which is the pathological hallmark of PD (Barrett et al., 2013; Sulzer et al., 2018). In clinical research, the contrast-to-noise ratio (CNR) and the volume of the NM-sensitive signal in the SNc have emerged as robust biomarkers that correlate significantly with both motor symptom severity and disease duration (Sulzer et al., 2018; Sasaki et al., 2006). Furthermore, NM-MRI serves as a critical tool for the differential diagnosis between idiopathic PD and atypical parkinsonian syndromes, offering high sensitivity in detecting early-stage depigmentation (Prasad et al., 2018). NM-MRI is carried out using a 3-T MAGNETOM Skyra equipment. Images are obtained through the 3D NM-sensitive T1-weighted turbo spin-echo sequence.

### 2.5 Biosample collection

**Figure 3:**
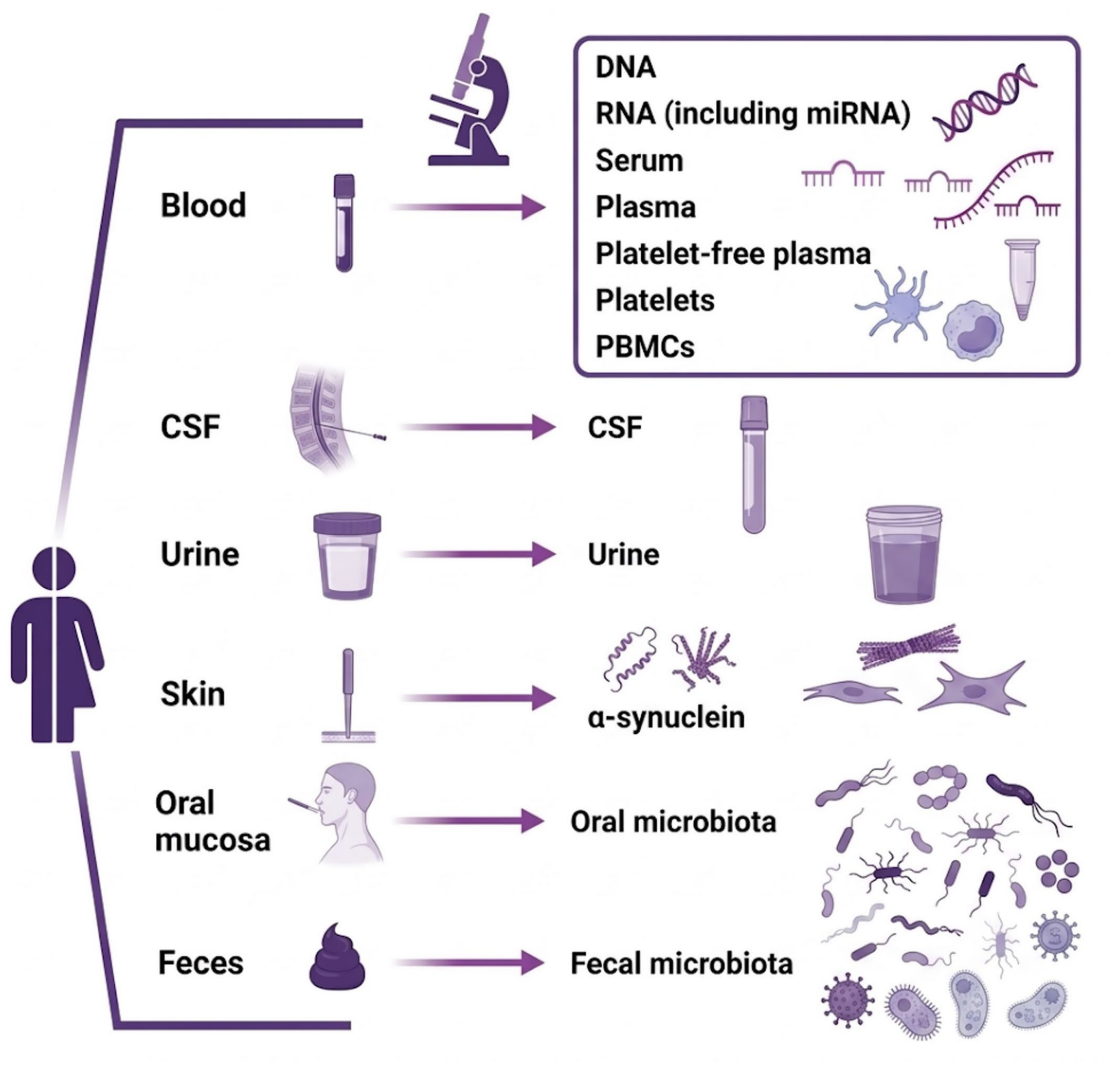
Biosamples collected during the VHIP study. The following biosamples are collected for each participant: (1) blood, (2) CSF, (3) urine, (4) skin, (5) oral mucosa, and (6) feces. From blood, DNA, RNA (including miRNA species), serum, plasma, platelets, and platelet-free plasma (including extracellular vesicles), and PBMCs are obtained. From skin biopsy, fibroblast cell lines are collected, and from olfactory mucosa, cells from epithelium. From oral mucosa and feces, microbiota from oral cavity and from feces are obtained.

Biosamples are collected for each participant during the first and second weeks: (1) blood, (2) CSF, (3) urine, (4) skin biopsy, (5) oral mucosa, and (6) feces (Figure 3). Briefly, five blood samples from regular venipunctures are collected and processed under fasting conditions. From these, DNA, RNA (including miRNA species), serum, plasma, platelets, and platelet-free plasma (including extracellular vesicles), and PBMCs are obtained for further analyses. In these fasting conditions, a mid-stream urine sample in a sterile urine cap is also collected. For CSF collection, lumbar puncture is an optional procedure performed first thing in the morning, unless contraindications exist (i.e., being treated with anticoagulant medication or clinical evidence of structural cerebral abnormalities). Under local anesthesia, a 20 G needle is carefully inserted between L3-L4 or L4-L5 vertebrae in the lower spine collecting up to 14 mL of CSF. For stool collection, a portable cooler is supplied at the first visit with the specific material and instructions necessary to avoid a contamination of the feces by skin or urinary microbiota, and to ensure the conservation of samples at the right temperature until they reach the laboratory. For oral mucosa sampling, a buccal swab and an Eppendorf tube for storage are provided in the portable cooler supplied during the first visit. Subjects are instructed to obtain the oral mucosa sample, in fasting conditions and without smoking for at least 1 h prior to sample collection, by gently brushing the buccal and sublingual mucosa bilaterally with the swab (these locations are optimal due to their proximity to the openings of the submandibular and parotid ducts). Immediately after sampling, feces and tubes with oral swabs are frozen at home at −20 °C until the next visit to the hospital (week 2), where they are stored frozen at −80 °C. For skin collection, 3 mm skin punch biopsy is performed under local anesthesia at C3–C5 cervical level twice; the first biopsy is used to establish fibroblast cell lines, and the second biopsy is frozen at −80 °C until further analysis. The following biosamples (i.e., feces, oral mucosa, olfactory mucosa, and skin biopsies) are processed directly at the Neurodegenerative Diseases Research Group of the Vall Hebron Research Institute (VHIR). The rest of biosamples are processed and stored at the Vall d’Hebron Biobank with their sample tracking system, sample processing system operation procedures, and standardized sample storage conditions being employed. All samples belong to the officially registered collection of the Bank of Neurodegenerative Pathologies within the Vall d’Hebron Biobank^1^.

### 2.6 Genotyping

#### 2.6.1 Genetic screening

A custom next-generation sequencing (NGS) panel targeting 33 genes with established or suspected relevance for PD is applied (Table 4). Genomic DNA is extracted from peripheral blood samples collected in EDTA tubes, using the blood DNA kit (0602, Danagene). A minimum of 100 ng of genomic DNA (≥2.9 ng/μL) is used for library preparation. Target-enriched libraries are prepared using the Cell3™ Target Library Preparation and Hybridisation & Capture Kits (Nonacus, Birmingham, United Kingdom), following the manufacturer’s instructions. Briefly, genomic DNA is enzymatically fragmented, end-repaired and A-tailed, followed by ligation of Illumina-compatible Unique Molecular Identifier (UMI) adapters (Nonacus). Libraries are purified using AMPure XP beads (Beckman Coulter) and amplified by 5 cycles of pre-capture PCR. Indexed libraries are pooled (1 μg total DNA), hybridized overnight (16 h) with a custom probe set, and captured using Dynabeads™ M-270 Streptavidin (Thermo Fisher Scientific). Post-capture amplification is performed with 14 PCR cycles, followed by purification with AMPure XP beads. Library quality and fragment size distribution are assessed using Qubit fluorometric quantification (Thermo Fisher Scientific) and QIAxcel electrophoresis (Qiagen). Sequencing is performed on a NextSeq 500/550 platform (Illumina) using a Mid Output v2.5 reagent kit (Illumina).

**Table 4:** NGS targeted panel, including 33 PD-linked genes.

Gene	Gene ID	Gene name
ATP13A2	23400	ATPase cation transporting 13A2
GBA1	2629	Glucosylceramidase beta 1
ATP6AP2	10159	ATPase H+ transporting accessory protein 2
LRRK2	120892	Leucine rich repeat kinase 2
VPS35	55737	VPS35 retromer complex component
VPS13C	54832	Vacuolar protein sorting 13 homolog C
SYNJ1	8867	Synaptojanin 1
DNAJC6	9829	DnaJ heat shock protein family (Hsp40) member C6
DNAJC13	23317	DnaJ heat shock protein family (Hsp40) member C13
TMEM230	29058	TMEM230 – transmembrane protein 230
PINK1	65018	PTEN induced kinase 1
PRKN	5071	Parkin RBR E3 ubiquitin protein ligase
PARK7	11315	Parkinsonism associated deglycase
FBXO7	25793	F-box protein 7
PLA2G6	8398	Phospholipase A2 group VI
SNCAIP	9627	Synuclein alpha interacting protein
SLC6A3	6531	Solute carrier family 6 member 3
CHCHD2	51142	Coiled-coil-helix-coiled-coil-helix domain containing 2
POLG	5428	DNA polymerase gamma, catalytic subunit
MAPT	4137	Microtubule associated protein tau
ATXN2	6311	Ataxin 2
TBP	6908	TATA-box binding protein
TAF1	6872	TATA-box binding protein associated factor 1
HTRA2	27429	HtrA serine peptidase 2
GLUD2	2747	Glutamate dehydrogenase 2
ATP1A3	478	ATPase Na+/K+ transporting subunit alpha 3
RIC3	79608	RIC3 acetylcholine receptor chaperone
ADH1C	126	Alcohol dehydrogenase 1C (class I), gamma polypeptide
GIGYF2	26058	GRB10 interacting GYF protein 2
EIF4G1	1981	Eukaryotic translation initiation factor 4 gamma 1
PODXL	5420	Podocalyxin like
SNCA	6622	Synuclein alpha
UCHL1	7345	Ubiquitin C-terminal hydrolase L1

FASTQ files were analyzed using a validated in-house bioinformatic pipeline. Reads were aligned to the human reference genome (GRCh38), followed by duplicate removal and base quality recalibration. Variant calling was annotated with publicly available databases (Varsome, ClinVar, NIH, and others). Quality control metrics included a minimum read depth of 20x, mean coverage of the target regions of 50x, with >98% of bases covered. Variants with a minor allele frequency (MAF) greater than 1% in population databases (gnomAD) were excluded as well as variants likely to be post-homopolymeric errors.

The targeted NGS panel was designed for the detection of Single Nucleotide Variants (SNVs) and small indels within the selected genes. As well as Copy Number Variants (CNVs), structural variants, and repeat expansions. Although the former were not systematically assessed and cannot be excluded. A limitation of the targeted NGS panel is its inability to reliably distinguish variants arising from the highly homologous *GBA1* pseudogene (*GBAP1*), potentially leading to reduced sensitivity for the detection of complex recombinant alleles and pseudogene-derived rearrangements. Variant interpretation was performed according to ACMG/AMP guidelines (Richards et al., 2015). Only variants classified as pathogenic or likely pathogenic were considered for clinical reporting in the current study, while Variants of Unknown Significance (VUS) were kept for further exploratory and functional analyses.

#### 2.6.2 ApoE gene genotyping

Using DNA extracted from peripheral blood samples, ApoE genotyping is conducted using Applied Biosystems real-time PCR platforms with commercially available TaqMan® DME probe-based genotyping assays (Applied Biosystems) for rs429358 and rs7412, with allelic discrimination performed using the manufacturer’s standardized fluorescence detection channels and software. This analysis is carried out at the National Genotyping Center (CeGen), Xenomic Medicine Group of the University of Santiago de Compostela (Spain).

### 2.7 Data collection, management, and statistical analysis

Data is collected and stored in an electronic Case Report Form (e-CRF) using the Research Electronic Data Capture System (REDCap) and will be transferred to a statistical package for subsequent analysis. Data unfit for storage in the eCRF, as imaging and metagenomic data, are kept on local secured servers at VHUH. All data are stored with polymorphic encryptions and pseudonyms, to guarantee the participants’ privacy on the one hand, and to enable data sharing on the other. Different statistical analysis such as descriptive studies, univariate studies, binary logistic regression including general linear models, will be performed depending on the type of objective.

*For primary longitudinal approach*: Linear mixed-effects models for continuous longitudinal outcomes (motor scores, imaging metrics, biomarker levels), with random intercept and slope per subject and fixed effects for group, time, and group × time interaction. Cox proportional hazards models for time-to-event outcomes (e.g., milestones such as motor complications, cognitive decline).

*Handling of missing data and dropouts*: Multiple imputation by chained equations (MICE, *m* = *X* (*m* at least equal to the % of missing data) will serve as the primary approach to handle missing data. Sensitivity analysis using patter-mixture models will be performed to assess robustness of results under a missing-not-at-random (MNAR) assumption, given the dropout in PD cohorts is unlikely to be completely random and may correlate with disease severity.

*Covariates systematically included*: All models will adjust for age, sex, disease duration, levodopa-equivalent daily dose (LEDD), and principal components of genetic ancestry derived from the NGS genotyping data. Educational level and smoking history will additionally be included as covariates for cognitive and sensory outcomes, respectively.

*Multiple comparison correction*: For the confirmatory pre-registered biomarker panels, the Benjamini-Hochberg false discovery rate (FDR) procedure will be applied at *q* < 0.05, balancing type I error control with statistical power in the contest of multiple correlated biomarkers. Family-wise error rate (FWER) correction will be reserved for the primary endpoint, defined as the rate of motor progression at year 2.5 and 5. Explanatory biomarker discovery analysis will report unadjusted *p*-values alongside FDR estimates, explicitly labeled as hypothesis-testing.

*Pre-registration*: Analysis plans for the confirmatory biomarker panel will be pre-registered and deposited before database lock, in line with open science principles and to avoid post-hoc analytical flexibility.

#### 2.7.1 Sample size

The sample size for this prospective cohort has been determined based on the primary objective of detecting clinically meaningful associations between fluid biomarkers (e.g., NfL, GFAP, lipidomics) and measures of dopaminergic degeneration, clinical progression, and multi-modal phenotypes in PD. Observational studies aiming to detect moderate correlations (effect size *r* ≈ 0.30) between continuous biomarkers and quantitative clinical or imaging outcomes typically require a sample size of approximately 80–100 subjects to achieve 80% power at an *α* level of 0.05 using two-tailed testing (Martínez et al., 2024; Teunissen and Khalil, 2012). Similar recommendations for cohort designs with biomarker associations have been outlined in recent neurodegenerative disease research, emphasizing the need for sufficient sample size to ensure reliable effect estimation and reduce false discovery rates (Marques et al., 2019).

In subgroup analyses—for example, comparing biomarker levels between genetic strata such as *GBA1* carriers versus non-carriers—detection of moderate effect sizes (Cohen’s *d* ≈ 0.5) generally necessitates at least 60–70 individuals per group (Mollenhauer et al., 2020). Given the relatively low prevalence of pathogenic variants in sporadic PD cohorts, achieving such subgroup sizes implies a larger overall recruitment target. To support both primary correlation analyses and stratified comparisons, a target cohort size of ~180–220 PD patients is considered sufficient. This range also accommodates longitudinal modeling, which benefits from repeated measures and retains power in the presence of attrition (Mollenhauer et al., 2020).

Notably, large multi-center cohort studies in PD and other neurodegenerative conditions have used similar or larger sample sizes to robustly characterize biomarker–phenotype relationships and validate prognostic signatures (Baek et al., 2021; Marek et al., 2018). Secondary and exploratory outcomes (e.g., lifestyle factors, comorbidities) are analyzed within this framework without adjusting the sample size specifically to each endpoint, consistent with modern multi-endpoint cohort methodology (Simuni et al., 2020).

### 2.8 Ethical considerations

The VHIP project is conducted in agreement with the standards for Good Clinical Practice, the fundamental ethical principles established in the Declaration of Helsinki for human studies and the Oviedo Convention, and the requirements established for research by the Spanish legislation. Potential participants to be included in this study receive detailed written and oral information about the study procedures, and verbal and signed informed consent is obtained from all recruited participants. We specifically ask all participants for their consent to the acquisition, analysis, and storage of biological samples. Access to samples can be requested by any researcher, from VHUH and from other institutions, after filling in a scientific request form and presenting the corresponding ethical permits to the Bank of Neurodegenerative Pathologies within the Vall d’Hebron Biobank. The VHIP cohort is based on blinded enrollment, and only clinically relevant results are disclosed. All the procedures described in the VHIP study have been approved by the local ethical Clinical Research Committee (PR(AG)170-2015 to guarantee compliance with the ethical and legal requirements of data acquisition, analysis, and storage.

## 3 Discussion

VHIP is a scientific effort focusing on deep phenotyping of *de novo* PD patients, subjects at risk with RBD, and asymptomatic MC with pathogenic *LRRK2* and *GBA1* variants, among others. With the future perspective to improve our understanding of early disease pathophysiology, to identify genetic and non-genetic modifiers of penetrance and expressivity, to contribute to disease subtype stratification, to accelerate disease-modifying therapeutic trials, and to identify and validate possible risk/diagnosis/prognosis biomarkers.

The clinical assessments and biosample collection scheme in VHIP are very extensive and cover multiple outcomes simultaneously to address the multi-dimensional nature of PD; including all the different aspects covered by other international efforts such as the Parkinson’s Progression Markers Initiative (PPMI), the LRRK2/Luebeck International Parkinson’s Disease (LIPAD) Study, the Dutch PARkinson Cohort (DUPARC), the Personalized Parkinson Project (PPP), the Luxembourg PD study and the Cincinnati Cohort Biomarker Program (CCBP) (Marek et al., 2018; Koprivica et al., 2000; Usnich et al., 2021; Boertien et al., 2020; Bloem et al., 2019; Sturchio et al., 2020). While other outstanding multicenter initiatives in Spain, such as the COPPADIS cohort (Santos-Garcia et al., 2016), have provided invaluable insights into Parkinson’s disease progression and clinical phenotypes, the VHIP study was specially designed to occupy a distinct and complementary niche. Unlike cohorts that encompass patients at various disease stages or on active dopaminergic therapy, VHIP restricts its recruitment strictly to treatment naïve, *de Novo* PD patients. Furthermore, the true scientific novelty of the VHIP cohort does not stem solely from its geographical setting, but rather from the powerful convergence of three specific features: (1) a dedicated genetic enrichment capturing mutation carriers from early stages, (2) a robust longitudinal follow-up, and (3) an extensive, multimodal biosampling repository (comprising blood fractions, cerebrospinal fluid, skin biopsies, fibroblasts, and gut microbiota). This multifaceted approach addresses critical gaps in early biomarker discovery that broad epidemiological or treated–patient cohorts cannot capture, positioning VHIP alongside major international benchmarks like the Parkinson’s Progression Markers Initiative (PPMI). The VHIP cohort shares with other studies focused on *de novo* PD (DUPARC, PPP and Luxembourg Parkinson’s Study) the advantage of single-center assessments, which provide a homogeneous approach to clinical and radiological data, as well as to the processing of biological samples. Concurrently, the study design and the assessment scales employed facilitate harmonization with data from international cohorts. In addition, the VHIP study will complement other studies focusing on pathogenic *LRRK2* and *GBA1* variants such as LIPAD and DUPARC, respectively. Finally, the nested studies planned to achieve the secondary objectives will provide a more in-depth phenotypic and molecular characterization especially of non-manifesting carriers of *LRRK2* and *GBA1* pathogenic variants and other PD-related pathogenic variants (Table 4). Additional studies are being planned, and potential funding opportunities are being explored, and we are open to collaboration with qualified researchers worldwide to address further topics and expand the objectives of this study. Importantly, the VHUH has an active Brain Bank and participants from the VHIP cohort are encouraged to join the Brain Donation Program^2^.

VHIP aims for a longitudinal follow-up of its study participants over a 10-15-year period, and beyond contingent on additional funding, providing a unique opportunity to link pre-diagnosis disturbances to PD development and to identify prognosis biomarkers, both essential points to improve disease treatment and management. In addition, considering the clinical and possibly etiological heterogeneity of PD, accurate patient stratification is necessary to advance our understanding of disease mechanisms and improve clinical trial design for future disease-modifying trials. Also, the identification and follow-up of MC at-risk that might fulfill the criteria for prodromal disease is critical to establishing a clinical-trial ready cohort for evaluating the impact of potential disease-modifying therapies, as well as allow the identification of protective and risk factors for phenoconversion to manifest PD.

One of the major limitations of this type of study is dropouts. As part of our retention effort, we are hosting annual Parkinson’s Day seminars since 2022 at VHUH for participants but also open to the general population. In addition, the study email address^3^ and website are used for regular recruitment updates, combined with an extensive information package on topics that scientists and participants consider relevant for people with PD and caregivers. Finally, pragmatic approaches such as streamlining data gathering and prioritizing biospecimen collection will be considered if important to retain subjects and minimize dropouts.

Overall, VHIP is an ambitious and challenging initiative that we anticipate will provide critical information regarding the natural history of PD, including motor and non-motor manifestations, imaging assessments and valuable data on genetic and molecular biomarkers. All of these will pave the way to develop a more precise and personalized disease management approach.

## Footnotes

1
https://vhir.vallhebron.com/en/research-support/biobank/collections/neurodegenerative-disorders-bank

2
https://vhir.vallhebron.com/en/research-support/biobank/collections/neurological-tissue-bank

3
parkinson@vhir.org
